# Gomesin peptides prevent proliferation and lead to the cell death of devil facial tumour disease cells

**DOI:** 10.1038/s41420-018-0030-0

**Published:** 2018-02-14

**Authors:** Manuel A. Fernandez-Rojo, Evelyne Deplazes, Sandy S. Pineda, Andreas Brust, Tano Marth, Patrick Wilhelm, Nick Martel, Grant A. Ramm, Ricardo L. Mancera, Paul F. Alewood, Gregory M. Woods, Katherine Belov, John J. Miles, Glenn F. King, Maria P. Ikonomopoulou

**Affiliations:** 1 QIMR Berghofer Medical Research Institute, Brisbane, QLD 4006 Australia; 2 Faculty of Medicine, The University of Queensland, Brisbane, QLD 4006 Australia; 3 Madrid Institute for Advanced Studies (IMDEA) in Food, CEI UAM+CSIC, Madrid, 28049 Spain; 4 School of Biomedical Sciences, Curtin Health Innovation Research Institute and Curtin Institute for Computation, Curtin University, Perth, WA 6845 Australia; 5 Institute for Molecular Bioscience, The University of Queensland, St Lucia, QLD 4072 Australia; 6 Menzies Institute for Medical Research, University of Tasmania, Hobart, Tasmania 7000 Australia; 7 School of Life and Environmental Sciences, University of Sydney, Sydney, NSW 2006 Australia; 8 Centre for Biodiscovery and Molecular Development of Therapeutics, Australian Institute of Tropical Health and Medicine, James Cook University, Cairns, 4870 Australia

## Abstract

The Tasmanian devil faces extinction due to devil facial tumour disease (DFTD), a highly transmittable clonal form of cancer without available treatment. In this study, we report the cell-autonomous antiproliferative and cytotoxic activities exhibited by the spider peptide gomesin (AgGom) and gomesin-like homologue (HiGom) in DFTD cells. Mechanistically, both peptides caused a significant reduction at G0/G1 phase, in correlation with an augmented expression of the cell cycle inhibitory proteins p53, p27, p21, necrosis, exacerbated generation of reactive oxygen species and diminished mitochondrial membrane potential, all hallmarks of cellular stress. The screening of a novel panel of AgGom-analogues revealed that, unlike changes in the hydrophobicity and electrostatic surface, the cytotoxic potential of the gomesin analogues in DFTD cells lies on specific arginine substitutions in the eight and nine positions and alanine replacement in three, five and 12 positions. In conclusion, the evidence supports gomesin as a potential antiproliferative compound against DFTD disease.

## Introduction

The Tasmanian devil (*Sarcophilus harrisii*) is the largest extant carnivorous marsupial^1^. The Tasmanian devil is an endangered Australian species, restricted to the island state of Tasmania^1^, and threatened with extinction due to a contagious and transmissible “parasitic” form of cancer known as devil facial tumour disease (DFTD)^2^, which has killed ~80% of the devil population since emerging in 1996^1^. Primary tumours appear on the face or inside the mouth and develop into large globular tumours that metastasize in a short period of time to internal organs and spread among individuals by biting during mating and territorial fighting^3^. Ultimately, DFTD leads to death within 3–6 months of the first appearance of clinical symptoms^4^. Although the disease emerged ~20 years ago in north eastern Tasmania, it now occupies most of the devil’s distribution with only small portions of the west and north western areas of the state remaining DFTD-free^1,4^. Modelling studies have estimated that without intervention the Tasmanian devil will extinct within the next 15–25 years^4^.

With no treatment available for DFTD, scientists maintain captive, disease-free breeding populations that are released into the wild. Major research efforts are focused on understanding the molecular mechanisms of DFTD and why tumour cells fail to stimulate an immune response in the Tasmanian Devil, including the dramatic downregulation of the expression of Major Histocompatibility Complex (MHC) Class I genes^5–9^. Previous trials to treat DFTD using human chemotherapeutic agents, such as vincristine, doxorubicin, and carboplatin have proven to be unsuccessful^10,11^. In this study, we postulated that spider peptides with vast pharmacology and activities ranging from analgesic to antimicrobial and antiproliferative properties^12^ may constitute a source of therapeutic leads against DFTD.

In order to test our hypothesis, we examined, for the first time *in vitro*, the anti-proliferative properties of the spider-venom peptide gomesin and its analogues as potential therapeutic lead candidates against DFTD. AgGom is an 18-residue peptide with documented anticancer activity^13,14^ that was first isolated from hemocytes of the South American mygalomorph spider *Acanthoscurria gomesiana*. Current studies from our lab have shown that AgGom and a gomesin analogue (HiGom) isolated from the Australian funnel-web spider *Hadronyche infensa* have similar antiproliferative properties (Ikonomopoulou et al., under review). This observation prompted us to characterise the cell-autonomous cytotoxic and anti-proliferative profile of gomesin in DFTD cells and in comparison, to non-transformed (healthy) Tasmanian devil fibroblasts (FIBS). In addition, we designed and screened a panel of gomesin analogues with amino acid modifications that were predicted to influence cell viability. Therefore, this study provides fundamental mechanistic insights into the antiproliferative properties of gomesin in DFTD.

## Results

### Gomesin peptides compromise DFTD4 cell viability

**Fig. 1:**
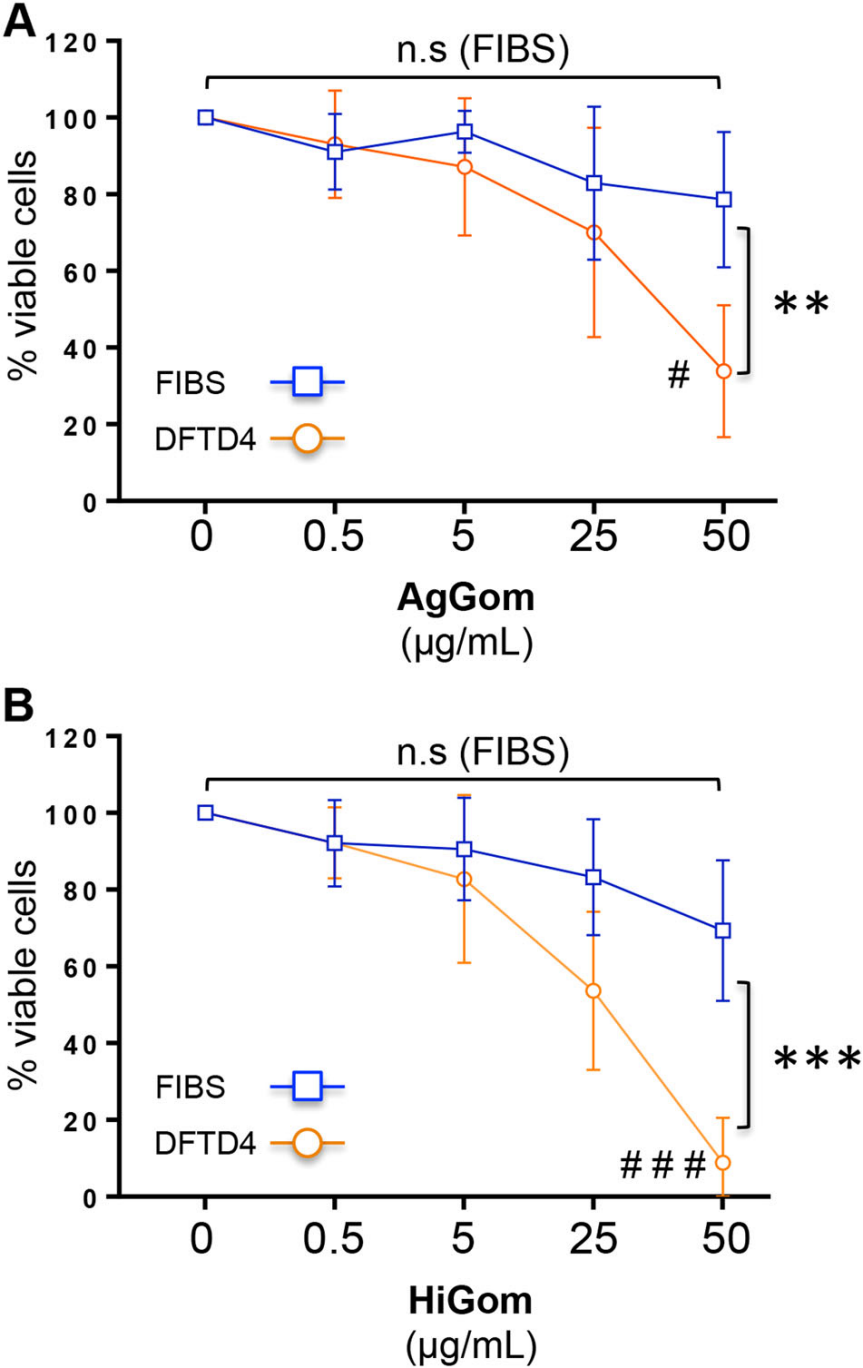
Gomesin compromises the viability of DFTD4 cells. Concentration-response data showing the effect of (**a**) AgGom and (**b**) HiGom on the viability of DFTD4 and FIBS cells treated with gomesin peptides for 48 h. Data are mean ± SEM. Experiments were performed in triplicate and are the result of three independent experiments. *P < 0.05, **P < 0.001, **P < 0.0001 (ANOVA: DFTD4 vs. FIBS) and ^#^*P* < 0.05, ^##^*P* < 0.001, ^###^*P* < 0.0001 (*t*-test: DFTD4, 0 vs. 50 μg/mL)

We used DFTD4 cell line as a DFTD cellular model to study the antiproliferative and apoptotic properties of gomesin peptides. First, we examined the potential cytotoxic and anti-proliferative effects of gomesin peptides by determining whether the viability of DFTD4 and FIBS cells was altered by 48 h exposure to either AgGom or HiGom. While at high concentrations (50 µg/mL) both AgGom and HiGom dramatically reduced the cell viability of DFTD4 cells, their deleterious effects on FIBS were not statistically significant (Fig. 1a, b). Most importantly, at lower concentrations, HiGom was more cytotoxic than AgGom to DFTD4 cells and it had negligible effects on FIBS ranging from 0.5 to 25 µg/mL (Fig. 1a, b). In addition, HiGom had an EC_50_ of 18.43 µg/mL while AgGom had an EC_50_ of 25.25 µg/mL. Hence, we concluded that HiGom is a better candidate for inhibiting progression of DFTD.

### AgGom and HiGom peptides reduce DFTD4 cells at G0/G1 phase, leading to cell death

**Fig. 2:**
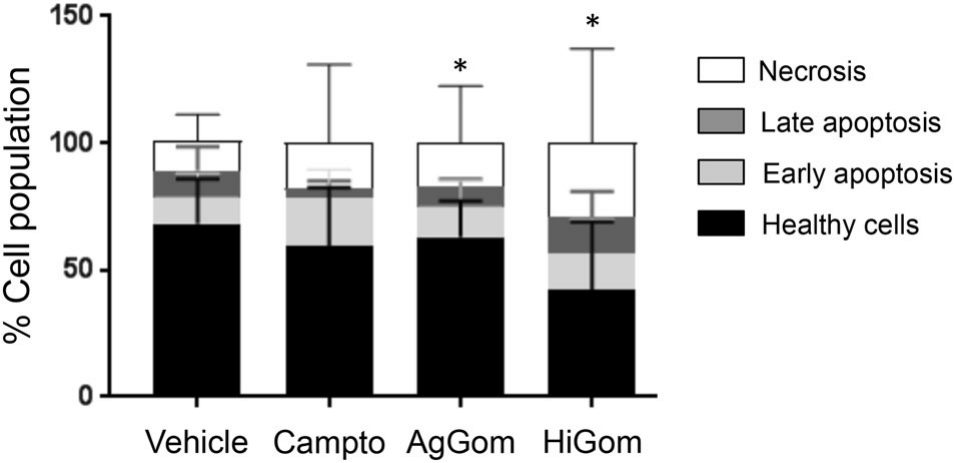
AgGom and HiGom induce necrosis of DFTD4 cells. AgGom and HiGom significantly increased necrosis of DFTD cells compared to untreated cells. Cells treated with camptothecin (Campto, 10 µM, 24 h) were used as a positive control (>30% late apoptosis). Data are mean ± SEM from three independent experiments. Statistics are relative to untreated cells and are represented as: *P < 0.05, **P < 0.001 (*t*-test or Wilcoxon test)

**Fig. 3:**
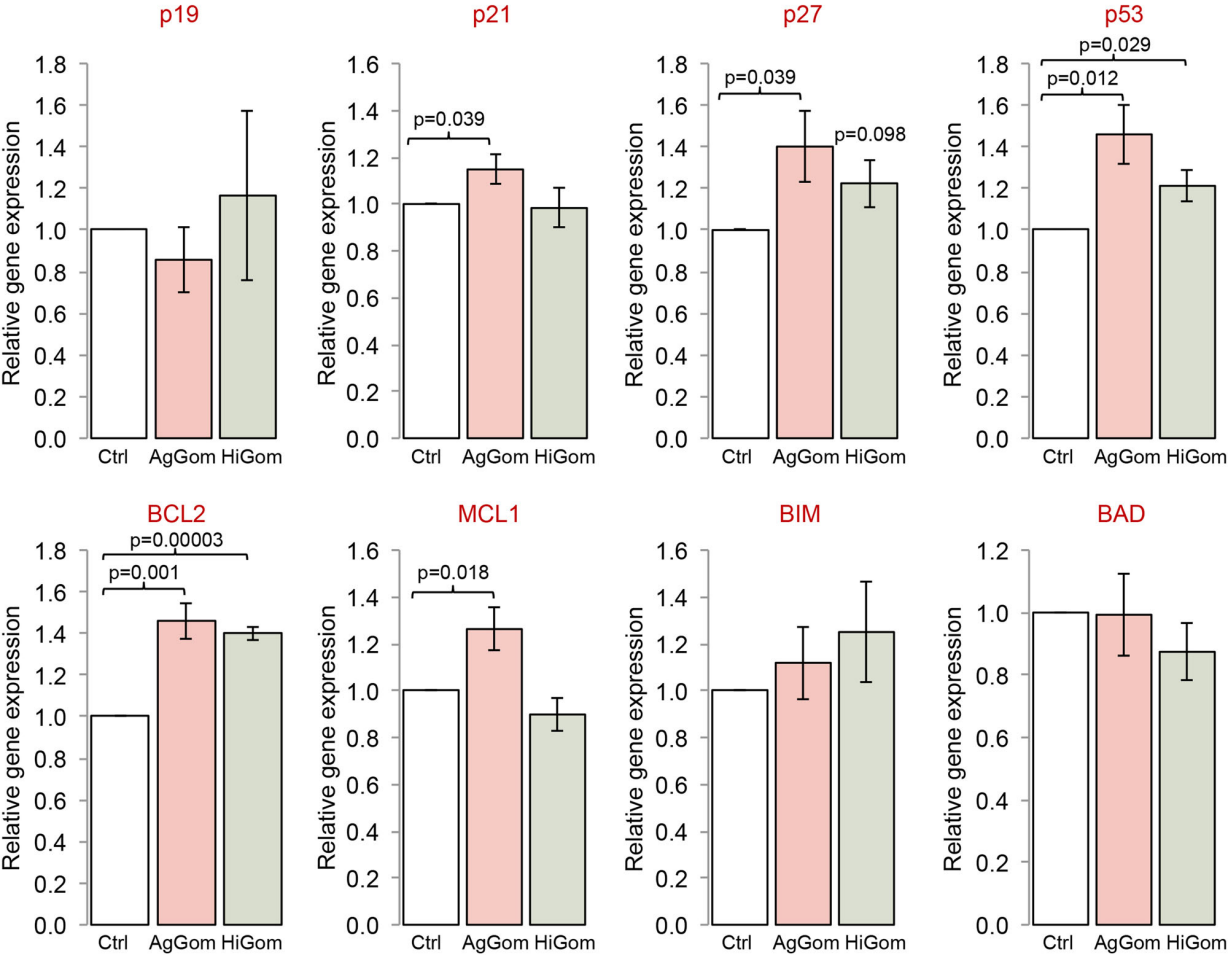
AgGom and HiGom stimulate the expression of cell cycle check-point, pro-survival and apoptosis-related genes in DFTD4 cells. Expression of cell cycle check-point genes (p16, p21, p27, and p53) and pro-survival/apoptosis-related (BCL2, MCL1, BAD, and BIM) genes in DFTD4 cells treated with 50 μg/mL AgGom (red bars) or HiGom (green bars) DFTD4. Gomesin-treated cells were compared to vehicle-treated cells (white bars) and are shown as mean ± SEM of three independent experiments. *P < 0.05 (*t*-test: AgGom or HiGom vs. vehicle)

Treatment of DFTD4 cells for 24 h with AgGom or HiGom induced a reduction of the cells in G0/G1 phase (Table 1). This correlated with elevated ratios of unprogrammed cell death (necrosis) of gomesin-treated DFTD4 cells in comparison to vehicle-treated cells (Fig. 2, *P* < 0.05). In addition, the early or late apoptotic cell ratio was not altered by gomesin treatments, while camptothesin, a well-known cell death promoter, was less potent than gomesin peptides (Fig. 2). To further comprehend the molecular mechanism that resulted in impaired cell cycle progression, cell viability, and stimulation of necrosis, we examined the gene expression of major cell cycle check-points (*p19*, *p21*, and *p27*) and proteins promoting cell death (*BIM*, *BAD*, and *p53*) but also the pro-survival proteins: *BCL2* and *MCL1*. AgGom and HiGom enhanced the expression of p53 and BCL2 (Fig. 3). Furthermore, AgGom showed a specific cellular signature in comparison to HiGom. Specifically, while p27 expression was elevated by both gomesin peptides, a statistically significant increase occurred only in AgGom-treated cells (Fig. 3). Moreover, AgGom-treated cells were characterised by induction of p21 and the pro-survival gene MCL1 (Fig. 3).

**Table 1:** The effect of AgGom and HiGom on cell cycle progression

	G0/G1	S-phase	G2/M
Untreated cells	56.57 ± 9.5	8.58 ± 0.7	18.4 ± 1.5
AgGom	39.9 ± 1.32*	11.47 ± 1.35	14.3 ± 0.9
HiGom	47.2 ± 0.66**	13.7 ± 1.9	13.3 ± 0.7

DFTD4 cells were treated with 50 μg/mL AgGom or HiGom for 24 h. Changes in cell cycle (G0/G1, S and G2/M phases) were analysed using FlowJo v10.06. Data are mean ± SEM and are the result of three independent experiments

*P  < 0.05, **P  < 0.001 (*t*-test or Wilcoxon test, relative to untreated cells).

### HiGom and AgGom enhance ROS content and diminish MMP in DFTD4 cells

**Fig. 4:**
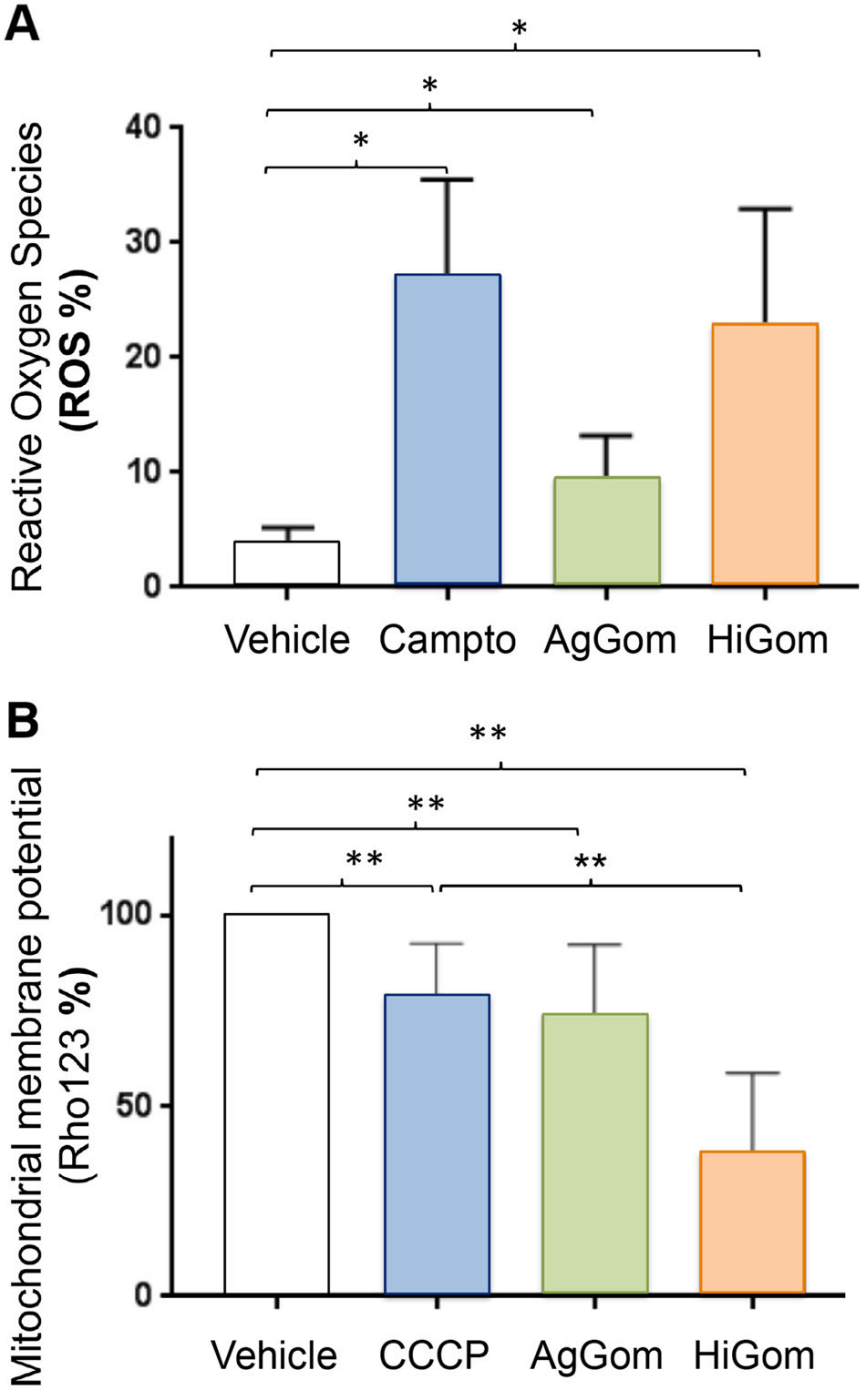
AgGom and HiGom increase ROS generation and reduce MMP in DFTD4 cells. **a** AgGom and HiGom (50 μg/mL, 24 h) increased ROS production in DFTD4 cells. Camptothecin (Campto, 10 µM, 24 h) was used as a positive control. **b** MMP in DFTD4 was significantly reduced after treatment with AgGom or HiGom. CCCP (0.5 mM, 12 h) was used as a positive control for MMP reduction. Data are mean ± SEM and are the result of three independent experiments. Statistics is relative to untreated cells and are represented as: *P < 0.05, **P < 0.001 (*t*-test or Wilcoxon test)

Excessive accumulation of reactive oxygen species (ROS), as well as loss-of-mitochondrial membrane potential (MMP) are molecular hallmarks of cancer cells. While normally p53 induces cell cycle arrest followed by apoptosis, as an exceptional situation, in mouse and human tumour cells enhanced activity of p53 has been linked to cell necrosis and elevated ROS^15^. Accordingly, elevated expression of p53 in response to gomesin treatments was accompanied by cellular stress. DFTD4 cells exposed to AgGom or HiGom for 24 h, exhibited a significant increase in ROS similar to those cells treated with our positive control of cellular stress, camptothecin^16^ (Fig. 4a). Moreover, AgGom and in a more profound manner HiGom (50 µg/mL, 24 h), caused a significant decrease in MMP in DFTD4 cells (Fig. 4b) in comparison to cells exposed to vehicle and to the positive control carbonyl cyanide 3-chlorophenylhydrazone (CCCP) (HiGom vs CCP; ANOVA, *F*_4,5_ = 15, *p* = 0.0038)^16^ (Fig. 4b).

### Branched and positively charged amino acids mediate gomesin cytotoxicity

**Fig. 5:**
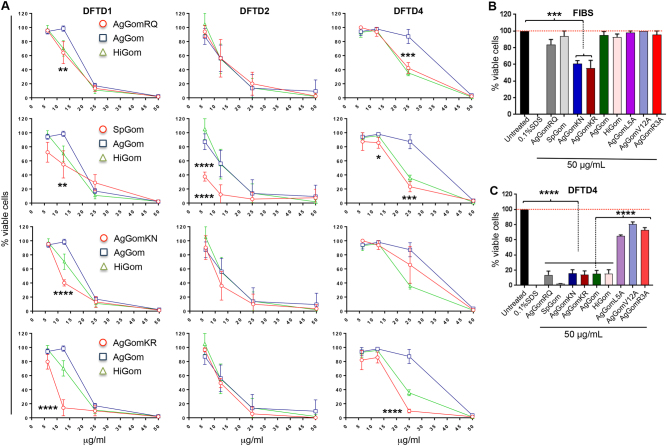
Analysis of the cytotoxic activity of novel gomesin analogues in DFTD cell lines. **a** Concentration-response in DFTD1, DFTD2, and DFTD4 cells exposed to 6.25, 12.50, 25, and 50 μg/mL of the analogues AgGomRQ, SpGom, AgGomKN, and AgGomKR for 48 h in comparison to AgGom and HiGom (**b**) FIBS and (**c**) DFTD4 cells treated for 48 h with 50 μg/mL of the analogues AgGomRQ, SpGom, AgGomKN, AgGomKR, AgGomL5A, AgGomV12A, and AgGomR3A in comparison to AgGom and HiGom. Data are shown as mean ± SEM and are the result of three independent experiments. Two Way-ANOVA was used to evaluate statistical difference between AgGom and the analogues, as well as ANOVA to determine differences between untreated cells and analogues (FIBS) and AgGom and analogues (DFTD4). ***P < 0.001, ****P < 0.0001

The results presented in the previous sections from functional and biochemical comparisons between HiGom (ZCRRLCY**RN**RCVTYCRGR) and AgGom (ZCRRLCY**KQ**RCVTYCRGR) implied that subtle modifications in the amino acid sequence (KQ8, 9RN, Table 2) constitute a feasible strategy to improve the anti-proliferative properties of gomesin against DFTD cells. Therefore, we designed a panel of analogues using single-amino acid substitutions of the two-residue motif that differentiates AgGom from HiGom (KQ vs. RN, Table 2). In addition to HiGom mutant, we synthesised AgGomRQ (K8R), AgGomKR (Q9R) and AgGomKN (Q9N). Moreover, based on alanine substitutions known to affect hydrophobicity and electrostatic surface potential of the peptide, we synthesised AgGomR3A, (R3A) AgGomL5A (L5A) and AgGomV12A (V12A). Finally, using the methodology previously described for the characterisation of HiGom (Ikonomopoulou et al., under review), we generated and tested a gomesin analogue isolated from the *Selenotypus plumpis* (SpGom; ZCRRICGRRRCFTYCRGR), whose sequence differs from AgGom by five residues (L5I, Y7G, K8R, Q9R, and V12F). In order to confirm the cytotoxic profile of gomesin and analogues, we tested them in DFTD4 and in two additional DFTD cell lines (i.e., DFTD1 and DFTD2). We observed that AgGomKN, AgGomKR, as well as SpGom exhibited higher anti-proliferative activity than AgGom and had minimal deleterious effects on FIBS cells (Fig. 5a–c). In addition, by examining the gomesin analogues, SpGom, AgGomKR, and HiGom, we observed that from each of the two amino acids that distinguished HiGom from AgGom, substitution of K or Q in positions 8 and 9 by arginine (R) are the more critical amino acid modifications driving and promoting the anti-proliferative properties of gomesin (Fig. 5a–c) (Table 2). Conversely, alanine substitutions in residues 3, 5, and 12 (AgGomR3A, AgGomL5A and AgGomV12A, respectively) eradicated the anti-proliferative activity of AgGom (Fig. 5c). Therefore, our mechanistic experimental approaches have identified key residues in AgGom that mediate its anti-proliferative and cytotoxic properties in DFTD cells.

**Table 2:** Amino acid sequences of AgGom, HiGom, and seven analogues

Analogue	Sequence
**AgGomRQ**	ZCRRLCY**RQ**RCVTYCRGR-*NH2*
**AgGomKN**	ZCRRLCY**KN**RCVTYCRGR-*NH2*
**SpGom**	ZCRR**I**C**GRR**RC**F**TYCRGR-*NH2*
**AgGomKR**	ZCRRLCY**KR**RCVTYCRGR-*NH2*
**AgGom**	ZCRRLCY**KQ**RCVTYCRGR-*NH2*
**HiGom**	ZCRRLCY**RN**RCVTYCRGR-*NH2*
**AgGomR3A**	ZC**A**RLCYKQRCVTYCRGR-*NH2*
**AgGomL5A**	ZCRR**A**CYKQRCVTYCRGR-*NH2*
**AgGomV12A**	ZCRRLCYKQRC**A**TYCRGR-*NH2*

In bold are the substituted from AgGom amino acids.

**Fig. 6:**
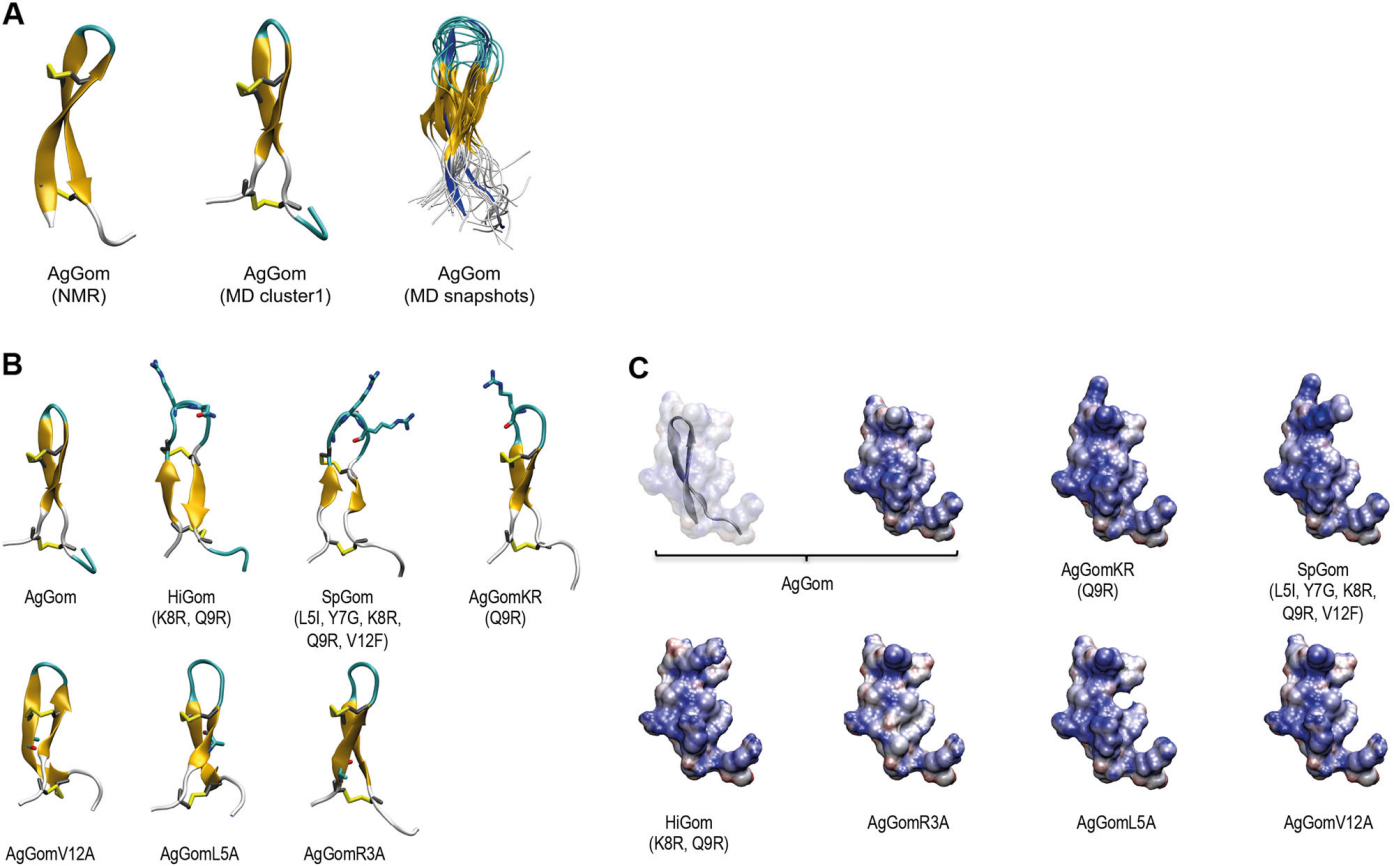
Representation of the structural conformation, hydrophobicity and electrostatic potential surface of native gomesin and analogues. **a** Structure of AgGom, including from left to right: NMR structure of native AgGom (PDB id 1KFP); representative structure of the predominant conformation of AgGom identified by clustering analysis of 3000 structures; overlay of 20 conformations from MD simulations of AgGom (the NMR structure is shown in dark blue as a reference). Structures are coloured according to secondary structure with β-strands in orange, turns in cyan and unstructured coils in white. Disulfide bonds are shown in yellow. **b** Structure of Gomesin variants from MD simulations. For each variant the structure shown is the predominant conformation identified by clustering analysis of 1200 structures. Structures are coloured according to secondary structure with β-strands in orange, turns in cyan, and unstructured coils in white. Disulfide bonds are shown in yellow. For variants, the side-chain of mutated residues is shown. **c** Electrostatic potential surfaces of native AgGom and gomesin variants. Structures are coloured from most electronegative (red) to most electropositive (blue)

We postulated that changes in the anti-proliferative properties of the different gomesin peptides might be a consequence of structural changes in the peptides or differences in conformational flexibility. At the conformational level previous studies using NMR revealed that AgGom adopts a two-stranded antiparallel β-sheet structure that is stabilised by two intra-strand disulfide bonds^17^. However, our analysis of 3000 structures from the combined trajectories clustered using a cutoff of 0.30 nm, an overlay of 20 conformations selected at random from the combined trajectory (Fig. 6a), as well as a root-mean square fluctuation analysis (RMSF) (data not shown), suggest that the level of conformational flexibility of AgGom is higher than expected from its NMR structure. As a result of this flexibility, the AgGom peptide can adopt a range of conformations in which the two strands are in different relative orientations (Fig. 6a). Despite these motions, the backbone–backbone hydrogen bonds characteristic of the β-sheet structure of AgGom are present for most of the simulation with distances between 0.16 and 0.25 nm. Only the hydrogen bond between pE1 and R3 is transient due to the flexibility of the C-terminus (Fig. 6a).

Comparative MD simulations of HiGom, AgGomKR, SpGom, AgGomL5A, AgGomR3A, and AgGomV12A with AgGom determined that amino acid substitutions in gomesin analogues do not confer structural modifications and different conformational dynamics that could explain their different cytotoxicity to DFTD cells (Fig. 6b). Comparison of the different structures shows that the C-terminus and the β-hairpin loop are the most flexible parts of the peptide and exhibit the same twisting and bending motions of the β-strands as seen in the AgGom simulations (Fig. 6b). RMSF analysis showed that SpGom, which exhibits a higher antiproliferative activity than AgGom and AgGomL5A, which lacks cytotoxicity properties, are more flexible than AgGom (Fig. 6b) while AgGomR3A is the least flexible and all other gomesin variants show RMSF profiles similar to AgGom. Therefore, changes in the conformational flexibility of the gomesin analogues do not either underlie their distinct anti-proliferative properties in DFTD cells.

Single-amino acid substitutions might also change the electrostatic potential surface of the peptide (Fig. 6c), which could affect their activity on DFTD cells, for example by altering the interaction of the peptide with the head groups of lipid molecules at the plasma membrane. Comparison of the different peptide variants shows that, as expected, substitutions of charged residues in AgGomKR, SpGom, and AgGomR3A have the largest effect on the electrostatic potential surface. Replacement of Q9 and/or K8 residues by arginine in AgGomKR and SpGom, respectively, increased the positive charge in the β-hairpin turn, resulting in a larger positively charged surface compared to AgGom. Conversely, substitutions outside the turn region, such as replacing R3 with alanine reduces the positive charge making the peptide surface more neutral compared to AgGom (Fig. 6c). However, amino acid substitutions in HiGom (K8R, Q9N) have no notable impact on the electrostatic potential surface despite increasing the cytotoxic activity of the peptide (Fig. 6c). Reduced electrostatic potential surface was also observed with either L5A or V12A substitutions in AgGomL5A and AgGomV12A, respectively (Fig. 6c). Nonetheless, and although not visible on the electrostatic potential surface, replacing L5 and V12 will reduce the hydrophobicity of the peptide, a property that is not observed in AgGomR3A, the other AgGom analogue lacking cytotoxic activity in DFTD cells. Moreover, when trying to explain the lack-of cytotoxic activity in AgGomR3A, AgGomL5A and AgGomV12A, we observed that L (leucine) and V (valine) are both branched and hydrophobic amino acids while R (arginine) is a positively charged amino acid. Therefore, changes in the electrostatic potential surface and type of amino acids do not either explain the cytotoxic activities of the different AgGom analogues Table 3.

**Table 3:** The EC_50_ values of AgGom, HiGom, and the cytotoxic gomesin analogues in three different DFTD cell lines

EC_50_ (µg/mL)	AgGomRQ	AgGomKN	SpGom	AgGomKR	AgGom	HiGom
DFTD1	14.54	11.5	12,5	**8.4**	20.4	14.96
DFTD2	13.87	10.62	**4.95**	12.47	12.29	13.06
DFTD4	23.08	27.4	18.35	**16.76**	20.41	14.96

The EC50s highlighted in bold are the best concentration values of the gomesin analogues observed in each DFTD cell line in comparison to AgGom and HiGom.

## Discussion

### Gomesin kills DFTD cells by necrosis

In this pioneer study, we examined for the first time, in our knowledge, the therapeutic potential of spider-venom peptides against DFTD. Our study provides a molecular and mechanistic characterisation of the mode of action of gomesin peptides to prevent the proliferation of DFTD cells. This is especially relevant since the Tasmanian devil population has declined by at least 80%^1^ and currently the therapeutic strategies to slow down the progression of DFTD in infective-devils have been ineffective. That was the case of vincristine, a chemotherapeutic agent used in human and veterinary medicine,^10^ as well as carboplatin and doxorubicin^11^. Only the immunomodulatory molecule imiquimod displayed apoptotic activity against cultured DFTD cells^7^. Therefore, novel compounds targeting DFTD cells are urgently needed. Regarding this, we believe that our data provide persuasive evidence of the specific-cytotoxic potential of gomesin peptides against DFTD cells. Indeed, ongoing studies in our lab corroborated and validated the cytotoxic properties exhibited by AgGom and HiGom on tumour cells (MM96L melanoma cells) in comparison to non-transform fibroblasts (Ikonomopoulou et al., under review). Moreover, and in agreement with the results obtained in melanoma MM96L cells (Ikonomopoulou et al., under review), HiGom seems to be a better therapeutic lead since it exhibited more dramatic antiproliferative and cytotoxic activity than AgGom in DFTD cells.

### Mechanism of gomesin-induced death in DFTD cells

The mode of action of AgGom and HiGom in DFTD cells shares molecular signatures with those observed in gomesin-treated human MM96L melanoma cells, including an increase of ROS and a reduction of MMP, hallmarks of cell necrosis and apoptosis in drug-induced cytotoxicity^18,19^. However, it is intriguing that unlike in gomesin-treated melanoma cells that suffer cell apoptosis (Ikonomopoulou et al., under review), gomesin-treatment leads to necrosis in DFTD cells. It is unclear whether both ROS generation and reduced MMP are causative factors of gomesin-induced necrosis or a consequence of gomesin cytotoxicity. However, and accordingly with the lack-of apoptosis, the expression of pro-apoptotic genes, such as BIM and BAD, both significantly upregulated in DFTD cells when exposed to imiquimod for 72 h^7^, is unchanged in gomesin-treated DFTD cells. Accordingly, gomesin induced a reduction of the cell population present in G0/G1 phase and elevated the expression of p53 and Bcl-2. While excessive and chronic increased expression of p53 may lead to apoptosis, p53, like Bcl-2, is originally considered as a pro-survival protein in response to deleterious environmental and cellular stress that cause cell cycle arrest and senescence^20^. In gomesin-treated DFTD cells, and although statistically significant, the stimulation of p53 and Bcl-2 expression is not higher than 1.5-fold the level observed in vehicle-treated DFTD cells, suggesting that in this case p53 and Bcl-2 may contribute to a pro-survival signalling to maintain cell integrity rather than causing apoptosis. Hence, our study suggests that gomesin exhibits a DFTD cell-specific and autonomous mode of action. Interestingly, AgGom and HiGom cytotoxic profiles demonstrated certain molecular differences. While it is possible that by stimulation and functional coordination of p53 and p27 both peptides regulate cell growth arrest, AgGom-treated DFTD cells reveal a unique signature involving upregulation of p21, another canonical cellular cell cycle check-point, and the anti-apoptotic gene MCL1. Hence, the data presented in this study suggest that there are mechanistic differences among gomesin peptides.

#### Characterisation of critical amino acid residues for gomesin-cytotoxic properties

The significance of this study is also underscored by the delineation of key residues responsible of gomesin cytotoxicity in DFTD cells, essential to further our understanding of the molecular mechanism of action of gomesin and the application of medicinal chemistry to design novel gomesin-based therapeutic strategies against DFTD. Critically, MD simulations and NMR determinations showed that the gain-of or lack-of anti-proliferative activities exhibited by the gomesin analogues were not mediated by changes in the peptide flexibility and/or conformations but as an intrinsic property of the different amino acid substitutions. It is feasible that like in other anti-proliferative or cell-penetrating peptides^21,22^, gomesin cytotoxic properties are governed by its interactions with the cell membrane. In linear homologues of gomesin it has been shown that membrane permeabilizing capacity is linked to the β-sheet motif^23–25^ and to hydrophobic residues, such as L5, Y7, and V12, which substitution results in reduced binding affinity to phospholipids and the ability to permeabilize membranes^26^. Our experiments using gomesin analogues showing substitutions of L5 and V12 by alanine are consistent with these studies. However, lack-of cytotoxic properties in the variant AgGomR3A despite its predicted increased binding to phospholipid vesicles and higher leakage than AgGom^26^ suggest that might exist other mechanisms mediating the deleterious effects on DFTD cells. Our studies of gomesin variants also confirm the important role of charged residues in the turn region. Indeed, these electrostatic interactions are believed to be an important factor in the selective cytotoxicity of anticancer peptides, such as AgGom^21,22^. In the SpGom and AgGomGR variants, that exhibit increased cytotoxicity in all three tested DFTD strain cell lines but minimal effects on FIBS, the positive electrostatic potential surface is larger than in AgGom and extends to the β-turn. We hypothesise that this may be related to the fact that an increased positive surface will enhance gomesin preference for negatively charged membranes^24^, which is the case of cancer cell membranes due to their high content in anionic lipids^26^.

In summary, taken together, the mechanistic characterisation of gomesin cytotoxic properties, as well as the design of powerful analogues and computational simulation approaches represent a significant step forward in understanding the molecular mechanism of gomesin-like peptides in order to apply medicinal chemistry and design gomesin-based therapeutic strategies against DFTD.

## Materials and methods

All reagents were obtained commercially and were used without further purification. Fmoc–protected l-amino acids Arg(Pbf), Asn(Trt), Cys(Trt), Gln(Trt), Gly, Leu, Lys(Boc), Phe, Thr(tBu), Tyr(tBu), and Val were purchased from IRIS Biotech (Marktredwitz, Germany), Bachem (Bubendorf, Switzerland), or ChemImpex (Wood Dale IL, USA). Unprotected l-pyroglutamic acid, dimethylsulfoxide (DMSO), triisopropylsilane (TIPS), diethyl ether, iodine, ascorbic acid and ammonium bicarbonate were purchased from Sigma-Aldrich (Castle Hill, Australia). Rink Amide polystyrene resin and *N*,*N*-diisopropylethylamine (DIEA) were purchased from Auspep (Tullamarine, Australia). Coupling reagent *O*-(6-Chlorobenzotriazol-1-yl)-*N,N,N’,N’*-tetramethyluronium hexafluoro-phosphate (HCTU) was purchased from ChemImpex (Wood Dale IL, USA), while *N*,*N*-dimethylformamide (DMF) and HPLC-grade acetonitrile were obtained from RCI Labscan (Bangkok, Thailand) and trifluoroacetic acid (TFA) and piperidine were purchase from Chem-Supply (Gillman, Australia).

Bacterial culture media from Bacto Laboratories (Mt Pritchard, Australia), and enzymes from Invitrogen and Life Technologies (Sydney, Australia). RPMI-1640 and AmnioMAX-C100 Basal Medium liquid and supplement were obtained from Invitrogen (Sydney, Australia). Annexin V-FITC Apoptosis detection kit was from BD Biosciences (San Diego, USA). Carboxy-H_2_DCFDA was from Invitrogen (Carlsbad, Australia). Rhodamine123, Propidium iodide (PI) and MTT kits were purchased from Sigma-Aldrich (Sydney, Australia), while primers were from Integrated DNA Technologies (Singapore Science Park II, Singapore).

### Selection of gomesin sequences for synthesis

AgGom and HiGom were synthesised as described below. We also synthesised peptides with modifications in the residues that differ between AgGom and HiGom and investigated whether these analogues would improve their anti-proliferative properties (Table 2, peptides AgGomRQ, AgGomKN, SpGom, and AgGomKR). We also substituted specific residues with alanine, following literature reports^26^, suggesting that these mutations alter binding affinity to lipids and lytic activity in AgGom (Table 2, AgGomL5A, AgGomV12A, and AgGomR3A).

### Chemical synthesis of gomesin peptides

The nine peptides were assembled on a 0.1-mmol scale using a Symphony automated peptide synthesizer (Protein Technologies, Tucson, USA) and a Rink amide polystyrene resin (loading 0.79 mmol/g) following the Fmoc/*t*Bu-solid phase peptide synthesis (SPPS) protocol. Chain assembly was performed in dimethylformamide (DMF) using 5-equivalents of Fmoc amino acid (AA)/*O*-(6-chlorobenzotriazol-1-yl)-*N,N,N’,N’*-tetramethyluronium hexafluorophosphate (HCTU) and DIPEA (AA/HCTU/DIPEA = 1:1:1) relative to resin loading for 2 × 30 min. Fmoc deprotection was achieved using 30% piperidine/DMF (1 × 1.5 min, then 1 × 4 min). Fmoc amino acids were side-chain protected as Arg(Pbf), Asn(Trt), Cys(Trt), Gln(Trt), Gly, Leu, Lys(Boc), Phe, Pyr, Thr(tBu), Tyr(tBu), and Val. Final cleavage and side-chain deprotection was accomplished using 90% TFA, 5% TIPS, and 5% H_2_O for 90 min at room temperature. The suspension was filtered, washed with TFA and the filtrate concentrated under steady N_2_ flow to a minimal amount. Subsequently, the peptide was precipitated and washed with cold Et_2_O. The precipitate was filtered off and then dissolved in 0.05% TFA in 50% MeCN/H_2_O and lyophilised. The reduced peptide was isolated by preparative reverse-phase (RP) HPLC and pure fractions were combined and lyophilised.

### Disulfide bond formation

Formation of the disulfide bonds was performed at room temperature in 0.1 M NH_4_HCO_3_ solution containing 10% DMSO (1 mg peptide/ml) at pH 8.3 for 18 h. Formation of the desired isoform was confirmed for all synthetic peptides using a combination of RP-HPLC and matrix-assisted laser desorption/ionisation mass spectrometry (MALDI-MS).

### Molecular modelling of gomesin peptides

#### Unrestrained molecular dynamics (MD) simulations of AgGom (native gomesin) and six variants

Details of the system setup and simulation parameters can be found in the supplementary material. Briefly, the NMR structure of gomesin (PDB-ID 1KFP^17^) was used as a starting structure. The peptide was modelled with a l-pyroglutamic acid (PCA) at the N-terminus and an amidated arginine (ART) at the C-terminus, and native disulfide bonds Cys2–Cys15 and Cys6–Cys11. For the six variants (HiGom, AgGomKR, SpGom, AgGomL5A, AgGomR3A, AgGomV12A), amino acid substitutions were introduced as outlined in Table 2. The peptide was solvated with water molecules and the charge was neutralised by adding Na^+^ ions. Further Na^+^ and Cl− ions were added to represent a final, physiological ionic strength of ~150 mM NaCl. AgGom was simulated for 500 ns in triplicate and the six peptide variants were each simulated for 300 ns in duplicate. All simulations were carried out using GROMACS 4.6.7^27^, in conjunction with the GROMOS 54A7 forcefield^28^. The temperature and pressure of the simulations system were maintained at 278 K and 1 bar for consistency with experimental conditions under which the NMR structure of gomesin (AgGom) was determined.

Simulations of a given peptide variant were combined for analysis into a single-data set containing 3000 structures for AgGom and 1200 structures for each variant. Conformational clustering, root-mean square deviation (RMSD) and root-mean square fluctuation (RMSF) calculations were carried out using GROMACS tools (Supplementary Table 1). Electrostatic potential surfaces were computed by solving the linearised Poisson–Boltzmann equation using APBS software (www.poissonboltzmann.org)^29^. All images were produced using VMD^30^. Details of the analysis can be found in the supplementary material.

### Cell lines

All DFTD (DFTD1, DFTD2, and DFTD4) and fibroblast (FIBS) cell lines were obtained from the Department of Primary Industries, Parks, Water, and Environment, State Government of Tasmania (DPIPWE) and were established as part of management plan and adhere to a standard operating practice. The DFTD1 cell line with catalogue number 3287 is derived from the tumour from a female devil (tag number: 06/2887) collected at St Marys, Eastern Tasmania in 2006. DFTD2 cell line with catalogue number 8961 was developed from a tumour from a male devil (tag number: 12/0625) collected at Kempton, Northern Tasmania in 2012. The DFTD4 cell line with catalogue number 4099 was developed from the tumour of a male devil (tag number: 07/0192) that was collected at Freycinet, east coast of Tasmania in 2007 and Fibroblasts cells (FIBS) with catalogue number 497 were obtained from a healthy devil male pup (tag number: 05/0962) held at Mount Pleasant, Southeastern of Tasmania in 2005.

### Cell culture

Devil Facial Tumor cancer cell lines, and the control Tasmanian Devil fibroblast (FIBS) healthy cell line were maintained in a humidified incubator at 35 °C and 5% CO_2_. The DFTD cell lines were cultured in RPMI-1640 medium supplemented with 10% FCS, and 2 mM Glutamax. The FIBS was grown in GIBCO AmnioMAX-C100 Basal Medium liquid, containing AmnioMAX-C100 supplement. Penicillin/streptomycin (PS) (100 U/ml each) was added to both media. The cells were passaged at ~90% confluency. Functional studies were performed with passages up to 20. All cell lines were mycoplasma free.

### Cell viability

Cell viability as an indicator of the number of viable and proliferative cells was measured by MTT according to the protocol of the manufacturer (Sigma-Aldrich, Australia). In brief, 8000 DFTD and 5000 FIBS cells/well were seeded in a 96-flat adherent microtiter well plate for 24 h to allow cell adhesion. Gomesin peptides and/or analogues were then added to the plates to measure MTT reduction. Plates were measured after 48 h at 540 nm absorbance in a microplate reader (BIOTEK PowerWave XS, USA). 0.1% SDS was used as a positive control (100% toxicity). A row of untreated cells was used to define 100% viability and blank wells containing only media were used to extract background. The concentration of gomesin peptides causing 50% inhibition (EC_50_) in DFTD and FIBS cells was determined using GraphPad Prism Software (Graphpad Inc, USA).

### Cell cycle

DFTD4 cells were synchronised by removing serum from the medium for ~24 h. Cells were then transferred into medium containing serum and treated with AgGom or HiGom at 50 µg/mL for 24 h. Cells were detached mechanically, washed with PBS and fixed with 70% ethanol for ~1 h at 4 °C. Ethanol was removed by centrifugation (453 g for 5 min) and cells were washed with PBS. Cells were treated with 5 µL of 10 µg/mL ribonuclease A to remove RNA contamination and further incubated for 1 h at 37 °C to allow release of low-molecular weight DNA. Cell pellets were stained with 10 µL of propidium iodide (PI) (1 mg/ml) and analysed at a maximum emission of 605 nm using a LSR Fortessa 5 analyser (BD Biosciences, San Diego, CA, US). Approximately 10,000 events were recorded and data were analysed using FlowJo software v10.06 (FlowJo, US).

### Cell apoptosis

Apoptosis in DFTD4 cells treated with AgGom or HiGom was measured using an Annexin V-FITC Apoptosis detection kit in combination with a Canto II high-throughput fluorescence-activated cell sorter (BD Biosciences, USA). Briefly, cells were seeded at a density of 100,000 cells/well in a round-bottom 96-well plate and treated with 50 µg/mL AgGom or HiGom for 24 h. The cells were collected mechanically, washed twice with PBS and once with wash buffer provided by the manufacturer. Cells were stained simultaneously with FITC-labelled annexin V and PI for 30 min in the dark and at room temperature before being analysed.

### Quantitative real time-PCR

Total RNA was extracted from both untreated (control) and gomesin-treated DFTD4 cells using an RNeasy kit (Qiagen, Australia) and 1 µg was reverse-transcribed into cDNA using SensiFast (Bioline, UK). Quantitative real time-PCR (qRT-PCR) to quantitate mRNA expression was performed on a LightCycler Instrument (Roche Molecular Biochemicals, Australia) with Tasmanian devil 18S used as the reference (housekeeper) gene. All primers are listed in Supplementary. Table 2.

### Reactive oxygen species

An Amplex Red Hydrogen Peroxidase assay kit (Invitrogen, Australia) was used to measure reactive oxygen species (ROS) generation in DFTD4 cells. A fluorescence probe that detects intracellular H_2_O_2_, Carboxy-H2DCFDA, was added 30 min prior to collecting cells to measure ROS production in DFTD4 cells that were previously treated with gomesin peptides at 50 µg/mL for 24 h. Fluorescent cells were washed twice with PBS prior to analysis on a FACSCalibur flow cytometer (BD Biosciences, USA) using excitation and emission wavelengths of 492 nm and 517 nm, respectively. Approximately 10,000 events were recorded per sample and the readout was analysed using FlowJo software v10.06 (FlowJo, USA).

### Mitochondrial membrane potential

We measured the mitochondrial membrane potential (MMP) using Rhodamine 123 (Rhod-123) (Sigma-Aldrich, Australia), a cationic dye that is localised in mitochondria. Loss-of-MMP results in loss-of-Rhod-123 fluorescence. Approximately 1 × 10^6^ DFTD4 cells treated with AgGom or HiGom at 50 µg/mL for 24 h were collected and resuspended in 0.1 ml of culture medium, stained with 10 µg/mL Rhod-123 for 30 min, and then washed with PBS. The intracellular concentration of Rho123 was determined immediately after by flow cytometry (BD Biosciences, USA) using an excitation wavelength of 488 nm. The data were analysed using FlowJo v10.06 (FlowJo, USA).

### Statistical analysis

All data are expressed as mean ± standard error of mean (SEM) of three independent experiments. Statistical analyses employed Student’s *t*-test or ANOVA for comparison between groups and control. *P* < 0.05 was considered statistically significant. A non-parametric Mann–Whitney test was used when populations within groups were not normally distributed. Calculations were performed with GraphPad Software (Graphpad Inc, USA).

## Electronic supplementary material

Supplementary Table 1

Supplementarty Table 2
