# Supplementary material for: Gomesin peptides prevent proliferation and lead to the cell death of devil facial tumour disease cells
Source: Cell Death Discov. 2018 Feb 14;4:19. doi: 10.1038/s41420-018-0030-0 (PMC5841354; doi:10.1038/s41420-018-0030-0)
Supplement: Supplementary file 1 — Supplementary Table 1 [file 41420_2018_30_MOESM1_ESM.docx]

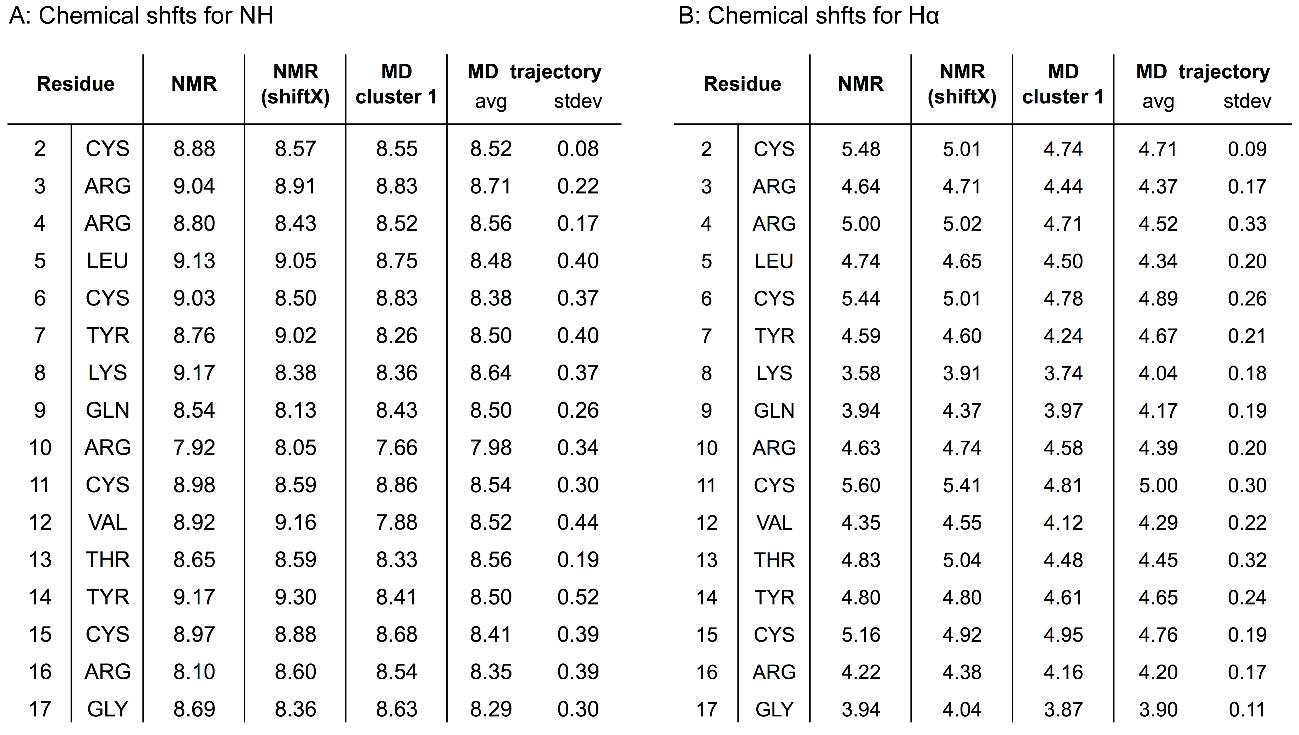


1. Mandard, N., Bulet, P., Caille, A., Daffre, S. & Vovelle, F. The solution structure of gomesin, an antimicrobial cysteine-rich peptide from the spider. *Eur J Biochem* **269**, 1190-8 (2002).
