# Supplementary material for: Gomesin peptides prevent proliferation and lead to the cell death of devil facial tumour disease cells
Source: Cell Death Discov. 2018 Feb 14;4:19. doi: 10.1038/s41420-018-0030-0 (PMC5841354; doi:10.1038/s41420-018-0030-0)
Supplement: Supplementary file 2 — Supplementarty Table 2 [file 41420_2018_30_MOESM2_ESM.docx]

| **Gene** | **Forward** | **Reverse** |
| --- | --- | --- |
| ***18S*** | AGCGGCTGAAGAAGATACGG | TTGGACACACCCACAGTACG |
| ***BCL2*** | GCGGATTGTGGCCTTCTTTG | AGTCATCCACAGGGCTATGC |
| ***MCL1*** | AGTTGTACGGGCAGTCCTTG | CCCCGTCACTGAACACATGA |
| ***BIM*** | CGTTTGCTACCAGATCCCCA | CACAACTCATAGGCGCTGGA |
| ***BAD*** | ATGAGCGACGAGTTCCACTG | CAAATTCCGCCCGAACCAAG |
| ***p53*** | AGCACAAAGATGCCTTGG | TCATAAGGGACGGACACAC |
| ***p19*** | TAGGGAAAGGAGGAGGATTG | CTGGATCCGACTGTCAGC |
| ***p21*** | GCGAGAGGAAGGGTCTATG | ATGGTAGAAATCCGTCATGC |
| ***p27*** | CCACGGATGATGTCTCAC | CTGAACTAGCATTGGGAGAAG |
